# Effect of dose and adjuvant on uptake of triclopyr and dicamba into *Pinus contorta* needles

**DOI:** 10.1002/pei3.10012

**Published:** 2020-04-25

**Authors:** Carol A. Rolando, Robyn E. Gaskin, David B. Horgan, Brian Richardson

**Affiliations:** ^1^ Scion Rotorua New Zealand; ^2^ Plant Protection Chemistry NZ Rotorua New Zealand; ^3^ Present address: Scion Rotorua New Zealand

**Keywords:** chemical control, methylated seed oil, translocation, uptake, wilding pine, woody weed management

## Abstract

Management of dense infestations of wilding *Pinus contorta* in New Zealand requires high doses of herbicides; 18 kg active ingredient (a.i.) ha^−1^ triclopyr and 5 kg a.i. ha^−1^ dicamba are used in combination with a complex mix of adjuvants (methylated seed oil, non‐ionic surfactant and ammonium sulfate) and other active ingredients. From the perspective of cost and environmental impact there is a need to reduce the complexity of this tank mix and the rates of active ingredients. Using radiolabelled herbicides, this study evaluated the effect of dose and adjuvants (crop oils, non‐ionic surfactants, and organosilicones) on needle injury and uptake of triclopyr and dicamba into *P. contorta* needles at 24 hr or 7 days after treatment (DAT). The uptake of triclopyr decreased significantly with increasing concentration (0.75%–6%) resulting in the highest uptake dose at the equivalent of 18 kg a.i. ha^−1^ triclopyr at 7 DAT. When applied at 18 kg a.i. ha^−1^, none of the adjuvants tested significantly increased the uptake of triclopyr (applied as Grazon^®^), with ~50% uptake occurring at 7 DAT. The uptake of dicamba (applied as Kamba^®^ at 5 kg a.i. and 10 kg a.i. ha^−1^) was significantly affected by dose and adjuvants. The uptake of dicamba applied at 5 kg a.i. ha^−1^ was low at 7 DAT with no adjuvant (31%); however, use of a methylated seed oil doubled the uptake. When triclopyr and dicamba were applied together, there was no evidence that either active ingredient negatively affected uptake of the other, with triclopyr enhancing uptake of dicamba. These results show potential to reduce the amount of herbicide used for conifer control without compromising efficacy.

## 1 INTRODUCTION

The spread of exotic conifers from commercial plantations, farm shelterbelts, and woodlots has been occurring for over 100 years in New Zealand, particularly the species *Pinus contorta* (Dougl.), *P. mugo* Turra, *P. nigra* Arnold, and *Pseudotsuga menziesii* (Mirb.) Franco. With an estimated 1.7 million hectares of land infested, these exotic conifers present a national‐scale weed problem in New Zealand (Ministry for Primary Industries, 2015). Recognition of wilding conifers as a significant land‐scale problem has been increasing over the last two decades (Ledgard, 2008; Ministry for Primary Industries, 2015), especially as they pose a significant threat to biodiversity, conservation and grazing potential on high country farms. The costs to stop and reduce the spread of these exotic conifers are, and will continue to be, high. However, there will be significant negative impacts on a range of social, economic, and environmental outcomes if spread continues. The need to effectively manage the spread of exotic conifers is not restricted to New Zealand, with South Africa, Sweden, Chile, and Brazil facing similar challenges with respect to the containment of invasive tree weeds (Engelmark et al., 2001; Ledgard, 2001; Rouget, Richardson, Milton, & Polakow, 2004; Simberloff et al., 2013).

There has been considerable research on herbicides for control of a range of infestation typologies of wilding conifers in New Zealand, extending back to the mid‐1970s (Davenhill & Preest, 1974; Gous, Raal, & Watt, 2014a, 2015; Gous, Watt, Richardson, & Kimberley, 2010; Langer, 1992; Preest, 1985; Ray & Davehill, 1991). For dense infestations of *Pinus* species, where canopy cover is estimated to exceed 80%, herbicides are generally aerially applied using helicopters fitted with a boom and nozzles that produce coarse droplets (volume mean diameter of about 350 µm), selected to minimize the risk of offsite spray drift (National Wilding Conifer Programme, 2018). While effective, the rates of herbicides used are high (Gous, Raal, & Watt, 2014b; Gous et al., 2010). The recommended spray mix, known locally as “TDPA” (Triclopyr, Dicamba, Aminopyralid, and Picloram), includes 18 kg a.i. triclopyr (3,5,6‐trichloro‐2‐pyridyloxyacetic acid), 5 kg a.i. dicamba (3,6‐dichloro‐2‐methoxybenzoic acid), 2 kg a.i. picloram (4‐amino‐3,5,6‐trichloropicolinic acid), 0.28 kg a.i. aminopyralid (4‐amino‐3,6‐dichloropyridine‐2‐carboxylic acid), 20 L of a methylated seed oil, 0.5 L of a lecithin blend, and 2.3 kg ammonium sulfate, all applied aerially in 400 L total volume (water) per hectare (Gous et al., 2015). The high application volume is required to ensure good coverage of foliage given the coarse droplet spectrum. While this treatment is effective and consistently achieves >90% control, from the perspectives of cost, environmental impacts, and operator health, concerns have been expressed as to the high rates of herbicides and complexity of the mix. Hence, there is an interest in evaluating whether more refined mixes could achieve the same outcome.

Both triclopyr and dicamba are selective, systemic post‐emergence, synthetic auxin herbicides that kill plants by inducing abnormal and uncontrollable growth (McBean, 2012). Like many other post‐emergence herbicides, the uptake of dicamba and triclopyr can be improved with the use of an adjuvant (Wang & Liu, 2007). Adjuvants have multiple functions in relation to pesticide efficacy and include spreaders, stickers, penetrants, and drift‐retardants that are either included as formulation components and/or as tank mix additives (Wang & Liu, 2007). Non‐ionic surfactants, organosilicones, synthetic and crop oil concentrates are adjuvants commonly used in herbicide mixes (Vanhaecke, 2000). Non‐ionic surfactants generally reduce the surface tension of the spray mix, among other effects (Wang & Liu, 2007). The crop oil concentrates typically increase the absorption and penetration of herbicides through the plant cuticle (Wang & Liu, 2007). They have also been shown to delay the crystallization of the herbicides on the leaf surface and reduce the volatile and photodegradative loss of some herbicides (Tse‐Seng, Kaben, & Thye‐San, 2009). Organosilicones generally lower surface tension and contact angle of water on leaves thereby promoting spreading on the leaf surface (Wang & Liu, 2007).

The problems presented by chemical control of landscape‐scale tree invasions highlight some fundamental questions pertaining to the strategy of control and prioritization of infestation typologies (isolated, scattered, and dense), as well as optimum application methods and their interaction with biological efficacy of herbicides (Forster & Kimberley, 2015; Forster, Pathan, Kimberley, Steele, & Gaskin, 2014; Nairn, Forster, & Leeuwen, 2016). The efficacy of foliage‐applied herbicide depends on many factors which include the physiochemical properties of the active ingredients, structures and concentration of surfactants, and leaf surface characteristics of the plant species (Stock, Edgerton, Gaskin, & Holloway, 1992). To optimize herbicide use, the factors driving or limiting the foliar uptake and translocation need to be understood. The opportunities to optimize synergism of active ingredients to increase biological efficacy also need investigation.

With reference to these issues and a need to refine the rates of herbicides used in operational control of isolated and dense infestations of *Pinus* species, a series of experiments were conducted on *P. contorta* to determine the effect of concentration, dose and adjuvants on needle injury, uptake and translocation of triclopyr (the key herbicide used for control of dense infestations of conifers). The key hypotheses tested were that uptake of triclopyr is proportional to concentration, that adjuvants will enhance triclopyr uptake and that translocation of triclopyr is proportional to uptake and inversely proportional to needle injury.

A similar set of experiments were also conducted to determine the effect of dicamba concentration and adjuvants on uptake of dicamba. This study was followed by an experiment that evaluated the interaction of triclopyr and dicamba when applied as a tank mix, with or without an adjuvant.

## 2 MATERIALS AND METHODS

### 2.1 Plant material, herbicides, and adjuvants

Wilding *P. contorta* seedlings (0.50–0.75 m) were collected from field sites between Taupo and Napier, on the North Island of New Zealand in June 2016. The seedlings were potted on location and brought back to the Scion nursery in Rotorua where they were placed outside and watered. These seedlings were used in all uptake and translocation experiments described below. All plants were equilibrated for 2 weeks before use, and treated, under controlled environment conditions as described for each experiment.

Two herbicide products and a number of adjuvants were used in these studies. In all experiments triclopyr was used as Grazon^®^ (600 g/L triclopyr butoxyethyl ester, Dow AgroSciences) and dicamba was used as Kamba^®^ (500 g/L dicamba as dimethylamine salt; Nufarm New Zealand). Details of adjuvants are included in Tables 1, 2, 3, 4 in appropriate sections.

**TABLE 1:** Uptake (as % of applied) of ^14^C‐triclopyr into *P. contorta* foliage at 1 DAT in three controlled experiments

Treatment	Experiment 1	Experiment 2	Experiment 3
Triclopyr @ 18 kg a.i. ha^−1^	26.5 ± 9 a	31.4 ± 8 a	30.1 ± 7 ab
+ MO^A^ 0.25%	25.2 ± 9 a		
+ MO^A^ 0.50%	21.1 ± 6 a	15.5 ± 4 bc	
+ MO^A^ 1.0%		11.7 ± 3 c	
+ AE^B^ 0.05%	23.6 ± 15 a		
+ AE^B^ 0.10%	26.1 ± 20 a		
+ MSO^C^ 0.5%			38.4 ± 6 a
+ MSO^C^1.0%			35.9 ± 6 a
+OS^D^ 0.15%		30.1 ± 10 a	22.7 ± 6 b
+OS^D^ 0.25%		22.5 ± 8 abc	31.2 ± 5 ab
+ operational adjuvants 4 kg NH_4_SO_4_ 20 L MSO^C^ 0.5 L lecithin blend^E^	22.6 ± 12 a	27.3 ± 9 ab	35.6 ± 9 a

Note

All treatments included triclopyr applied at 18 kg a.i. ha^−1^ in the equivalent of 400 L/ha water with or without adjuvant. Standard deviation shown after means. Means followed by different letters within each experiment are significantly different at *p* < .05.

Abbreviations: AE, alcohol ethoxylate; DAT, days after treatment; MO, mineral oil; MSO, methylated seed oil; OS, Organosilicone.

^A^ Uptake™ Spaying oil, Dow AgroSciences, New Zealand.

^B^ Actiwett^®^, Etec Crop Solutions Ltd.

^C^ Kwickin Oil, STT New Zealand.

^D^ Du Wett^®^, Etec Crop Solutions Ltd.

^E^ Li 700^®^, Etec Crop Solutions, New Zealand.

**TABLE 2:** Uptake and translocation of ^14^C‐triclopyr at 1 DAT and 7 DAT from treatments applied in the equivalent of 400 L/ha water to *P. contorta* needles

Treatment	Uptake %		Translocation of absorbed %	Total amount translocated (kg/ha)	Injury score (1–5)
	1 DAT	7 DAT	7 DAT	7 DAT	7 DAT
18 kg a.i. ha^−1^	39.8	47.8 ± 4 ab	91.4 ± 5 a	7.9	0.5
18 kg a.i. ha^−1^ + 5% MSO^A^	51.0	52.6 ± 6 a	77.2 ± 7 a	7.3	2.6
18 kg a.i. ha^−1^ + operational adjuvants^B^	15.6	31.5 ± 5 b	47.3 ± 6 b	2.7	2.8

Note

Needle injury at 7 DAT also shown. Means followed by different letters are significantly different at *p* < .05.

^A^ Punch^®^, modified vegetable oil and surfactant blend, Etec Crop Solutions, Ltd.

^B^ 20 L/ha MSO, 0.5 L/ha lecithin blend, and 4 kg/ha ammonium sulfate.

**TABLE 3:** Uptake of ^14^C‐dicamba and needle injury, at 1 DAT and 7 DAT, from treatments applied to *P. contorta* foliage in the equivalent of 400 L/ha water

Treatment	Uptake (%)		Injury score	
	1 DAT	7 DAT	1 DAT	7 DAT
Dicamba: 5 kg a.i. ha^−1^	2.0	31.3 ± 3 c	0	0
Dicamba: 10 kg a.i. ha^−1^	—	14.3 ± 3 d	—	0.38
Dicamba: 5 kg a.i. ha^−1^ + 5% MSO^A^	—	66.1 ± 3 b	—	3.13
Dicamba: 5 kg a.i. ha^−1^ + operational mix adjuvants^B^	47.7	78.6 ± 3 a	2.13	3.69

Note

Means followed by different letters are significantly different at *p* < .05.

^A^ Punch^®^, modified vegetable oil and surfactant blend, Etec Crop Solutions.

^B^ 20 L/ha MSO, 0.5 L/ha lecithin blend, 4 kg/ha ammonium sulfate.

**TABLE 4:** Uptake of ^14^C‐triclopyr and ^14^C‐dicamba at 1 DAT, and needle injury at 1 DAT and 7 DAT from treatments applied to *P. contorta* foliage in equivalent of 400 L/ha water

Treatment	kg a.i.ha^−1^ + % adj	Uptake (%)		Injury score (0–5)	
		Dicamba	Triclopyr	1 DAT	7 DAT
Dicamba	5	30.8 c	—	0.3	0.3
Triclopyr	15	—	25.8 a	0.0	0.1
Dicamba + triclopyr	5 + 15	46.9 b	24.3 a	0.7	1.0
Dicamba + triclopyr + MSO^A^	5 + 15 + 5%	60.9 a	37.7 a	2.6	3.4
Dicamba + triclopyr	5 + 10	49.2 ab	34.1 a	0.4	1.2
Dicamba + triclopyr + MSO^A^	5 + 10 + 5%	62.1 a	28.8 a	2.1	3.1
Dicamba + triclopyr	10 + 10	42.9 bc	—	1.0	1.9
Dicamba + triclopyr + adj^B^	5 + 18	54.6 ab	(31.5)	2.6	3.6

Note

Means within a followed by different letter are significantly different at *p* < .05.

^A^ Punch^®^, modified vegetable oil and surfactant blend, Etec Crop Solutions.

^B^ Adjuvants used in the operational TDPA mix, see Materials and Methods.

### 2.2 Method used for all uptake and translocation assessments

The quantification of uptake and translocation of triclopyr and dicamba in all experiments described below was made using radiolabelled active ingredients. For all treatments, the radiolabelled active ingredient, either ^14^C‐triclopyr butoxy ethyl ester (specific activity = 481 MBq/mmol) or ^14^C‐dicamba (specific activity = 1742 MBq/mmol), was added to the test formulations at a concentration of 4 MBq/µL. To achieve this, appropriate volumes of radiotracer solution were dispensed into microvials and the carrier solvent (dichloromethane) evaporated under nitrogen. Concentrated solutions of product (either Grazon^®^ or Kamba^®^) were dispensed on top of the radiotracer, sonicated for 2 min and allowed to equilibrate at room temperature for 1 hr. These were then diluted with appropriate concentrations of formulants to prepare the treatments at the required concentration and specific activity, and checked to ensure the solutions were solubilized and homogenous prior to use. For all experiments, treatments were applied via droplet (0.24 µl) application with a 10 µl microsyringe to the surface of mature *P. contorta* needles on six to eight replicate plants, either randomly or specifically to the inner and/or outer surface of needles as required for the test. Droplet density on foliage simulated a spray application volume of 400 L/ha. Numbers of plants included in uptake and translocation experiments were limited by the size of the growth chambers maintaining controlled environmental conditions. A droplet size of 0.24 µl was used for all experiments as this is the smallest, reliably reproducible volume able to be dispensed from a 10 µl syringe with a repeating dispenser.

At 24 hr after treatment (HAT) or 7 DAT, the surface of treated needles in all treatments was washed with 100% acetone (4 ml). This was diluted with water (1:1) and incorporated into scintillation solution (13 ml ACS, Amersham) to quantify unabsorbed radiolabelled active ingredient. Uptake was determined as the quantity of radiotracer applied minus that recovered in washes. For determination of translocation, treated needles from the 7‐day harvest were stored frozen until combusted in a Harvey Oxidiser to quantify radiolabelled herbicide (triclopyr or dicamba) present in the treated needle. The amount of radiolabelled active ingredient not recovered in surface washes or treated needles was determined as translocated. Any loss due to volatilization was not accounted for. Translocation is reported as a percentage of the amount of herbicide absorbed into the plant.

Where required, the effect of herbicide treatments on needle health was assessed at 24 HAT and/or 7 DAT by scoring injury (browning) on a scale of 0–5, where 0 = no injury and 5 = total brownout of the treated needles.

### 2.3 Quantification of droplet spread

An assessment of the effect of pine needle surface (inner vs. outer) on droplet spread was made prior to all experiments. Solutions of triclopyr at rates equivalent to 18 kg a.i. ha^−1^ in 400 L water (or 4.5%) were prepared as described above but with the addition of a water‐soluble fluorescent dye (Blankophore P, Bayer) at a concentration of 0.5% w/v. Twelve droplets (0.24 µl) of each solution were applied to the inner and outer surfaces of each of three random pine needles selected from different test plants. Droplets were left to spread for 10 min after which needles were illuminated with ultraviolet light and photographed. The area of droplet spread was determined using image analysis software (V++™, Digital Optics Ltd).

### 2.4 The effect of triclopyr concentration on uptake

The effect of concentration (or dose) on the uptake of triclopyr was determined by the addition of radiolabelled triclopyr to spray formulations of different concentrations (0, 0.75%, 1.5%, 3%, 4.5%, and 6.0%) or use rates (0, 3, 6, 12, 18, and 24 kg a.i. ha^−1^). All treatments were applied at the equivalent of 400 L/ha total spray volume and replicated on the inner and outer surfaces (five droplets on each surface) of mature needles (previous year's growth) on six separate potted *P. contorta* plants. Plants were maintained in a controlled environment (26/16°C day/night), 14 hr day length and relative humidity of 50%. Needle injury was determined on all treated plants at 1 DAT and 7 DAT.

### 2.5 The effect of adjuvants on uptake of triclopyr

Three controlled experiments (referred to as Experiments 1, 2, and 3) were carried out to determine the effect of four classes of adjuvants on the uptake of triclopyr applied at 18 kg a.i. ha^−1^ in the equivalent of 400 L/ha total spray volume. These experiments were sequential and reflect refinements in choice of adjuvant and rate as results became apparent. The adjuvants tested included an organosilicone (OS) at 0.15% and 0.25% (Du Wett, Etec Ltd), an alcohol ethoxylate (AE) at 0.05% and 0.1% (Actiwett, Etect Ltd), a mineral oil (MO) at 0.25%, 0.5% and 1% (Uptake Spraying oil, Dow AgroSciences), and a methylated seed oil (MSO) at 0.5% and 1% (Punch, STT New Zealand). All experiments included two treatments: (a) triclopyr applied at 18 kg a.i. ha^−1^ as Grazon^®^ only (no adjuvant) and (b) triclopyr applied at 18 kg a.i. ha^−1^ as Grazon^®^ with the adjuvants used in current operational control standard, or TDPA mix: 20 L MSO, 0.5 L lecithin blend, and 4 kg ammonium sulfate (Table 1). For all experiments the effect of the adjuvants on triclopyr uptake was assessed at 24 HAT as this was estimated to be the period most likely to reflect that which would occur infield where exposure to sunlight and or rain could be mean rapid degradation or wash‐off of the active ingredient. Treatments were applied to the surface of mature needles on six separate replicate plants kept under controlled environment conditions of 26/16°C day/night, 50% relative humidity, and 14 hr day length.

### 2.6 The relationship between needle injury, uptake, and translocation of triclopyr

A study was carried out to determine the effect of needle injury on uptake and translocation of triclopyr in *P. contorta* needles when applied at 18 kg a.i. ha^−1^ as Grazon^®^ with: (a) no adjuvant, (b) an MSO (5%), and (c) the adjuvants included in the operational “TDPA” mix (Table 2). Assessments of needle injury, uptake, and translocation of ^14^C‐triclopyr were made at 24 HAT and 7 DAT. Treatments were applied to the surface of mature needles on eight separate replicate plants kept under controlled environment conditions of 26/18°C day/night, 50% relative humidity, and 14 hr day length.

### 2.7 The effect of concentration and adjuvants on needle injury and uptake of dicamba

Treatments are described in Table 4. Treatments were applied to the mature needles of eight separate *P. contorta* plants kept under controlled environment conditions as described previously.

### 2.8 The uptake of triclopyr and dicamba when applied as a tank mix

The final study assessed the interaction between triclopyr and dicamba when applied as a tank mix (as is currently recommended for the operational control of dense infestations of *P. contorta*; Table 4). For this study, duplicate solutions containing either ^14^C‐triclopyr butoxy ethyl ester or ^14^C‐dicamba were applied as separate treatments on the same replicate trees at the same time, with all other experimental procedures equivalent to that described above. The uptake (7 DAT) of dicamba and triclopyr was determined alone at 5 or 15 kg a.i. ha^−1^, respectively, and then in ratios of 1:3, 1:2, and 1:1, with or without an MSO. Needle injury was scored at 1 DAT and at 7 DAT. Treatments were applied to the mature needles of eight separate *P. contorta* plants kept under controlled environment conditions as described previously.

### 2.9 Analysis

Data for each experiment were analyzed using the general linear model procedure (Minitab 19, Minitab LLC). Treatment was incorporated into the model as a fixed effect. Where necessary, data were transformed (log or square‐root) prior to analyses to meet the assumptions of normality (Kolmogorov–Smirnoff test) and homogeneity of variance (Bartlett's test) required for a valid ANOVA. Where the overall *F* test was significant (*p* < .05), treatment means comparisons were made using Tukey's test. Where uptake and translocation were determined at 1 DAT and 7 DAT, only uptake at 7 DAT has been analyzed. Data shown in tables are percentage means (untransformed data); however, the letters indicating significance are based on the analysis performed on transformed data (where necessary).

Linear regression (Minitab 19, Minitab LLC) was used to define the relationships between the concentration of triclopyr and uptake (with or without MO).

## 3 RESULTS

### 3.1 Quantification of droplet spread

**FIGURE 1:**
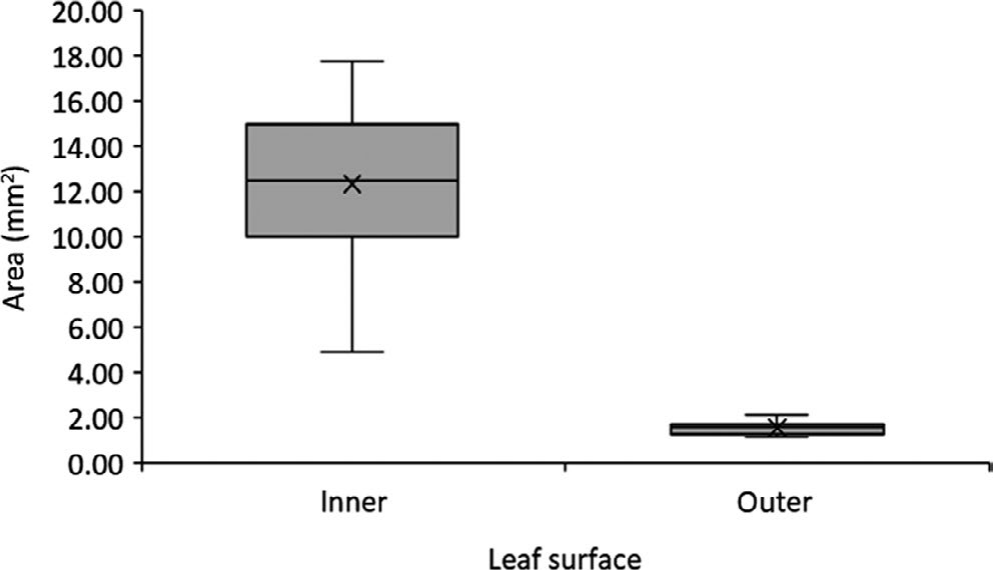
Droplet spread on inner and outer needle surfaces of *P. contorta* needles when applied at 18 kg a.i. ha^−1^ in the equivalent of 400 L total volume water

There was a significant difference (Students *t* Test; *T*‐value = 9.81, DF = 11; *p* < .05) in spread of droplets, with significantly more spread on inner needle surfaces (Figure 1). In all further experiments, treatment droplets were placed equally on inner and outer needle surfaces.

### 3.2 The effect of concentration on uptake of triclopyr

**FIGURE 2:**
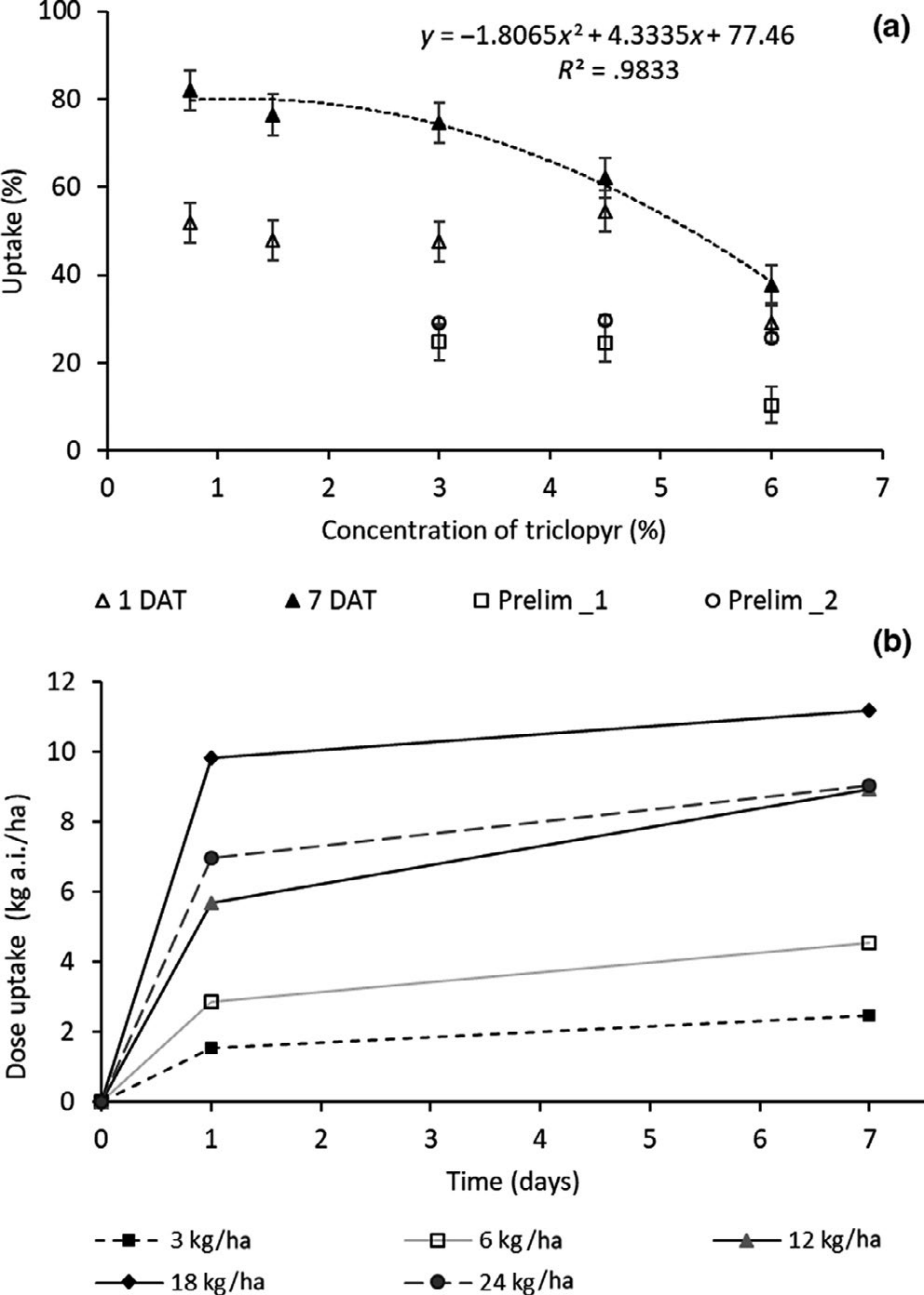
(a and b) Uptake of triclopyr at 1 DAT and 7 DAT applied as Grazon^®^ shown as a function of (a) concentration and (b) dose. The points on (a) labeled Prelim 1 and 2 indicate outcomes of similar previous preliminary studies

At 1 DAT there were no significant differences in triclopyr uptake, as a percentage of the amount applied, over the concentration range of 0.75%–4.5% (equivalent to dose range of 3 kg to 18 kg a.i. ha^−1^), although the trend was for decreased uptake as concentration increased (Figure 2a). When applied at 6%, or 24 kg a.i. ha^−1^, uptake of triclopyr at 1 DAT significantly decreased as a percentage of active ingredient applied (Figure 2a). While a high proportion of triclopyr uptake occurred within 1 DAT, uptake continued up to 7 DAT, especially at the lower concentrations tested (Figure 2b). Lower uptake at higher concentrations was also observed in similar, preliminary studies with triclopyr covering a concentration range of 3%–6% (Figure 2a). The highest uptake of triclopyr into the needles was achieved with the 18 kg a.i. ha^−1^ treatment (Figure 2b). Injury to needles was observed to increase with dose but was generally insignificant, with the highest injury score of 1.7 observed for the highest dose (24 kg a.i. ha^−1^) or 6% (data not shown).

### 3.3 The effect of adjuvants on uptake of triclopyr

In all experiments a high variability between replicate treatments was observed likely caused by the large natural differences occurring in the non‐clonal wilding trees used in the study.

**FIGURE 3:**
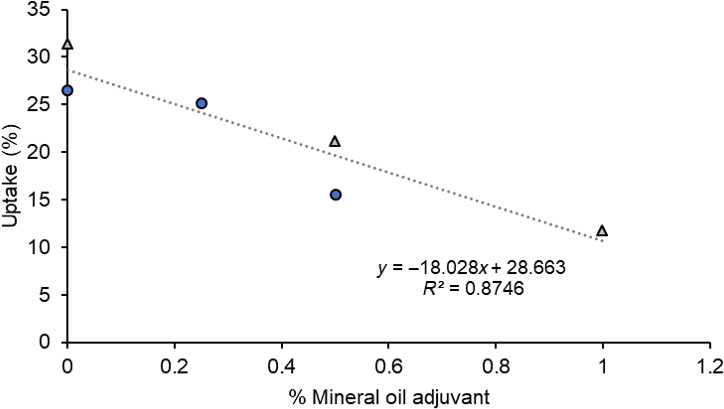
Uptake of triclopyr at 18 kg a.i. ha^−1^ in 400 L water, or 4.5%, formulated as Grazon^®^ as a function of inclusion of a mineral oil adjuvant in the mix. Symbols represent data from Experiment 1 (circles) and Experiment 2 (triangles)

In Experiment 1, triclopyr uptake ranged from 21% to 26% (Table 1). Although the highest uptake was obtained with triclopyr alone and the lowest with a MO adjuvant, there were no significant differences between treatments, including the operational treatment (DF = 6; *F* = 0.13; *p* = .992). In Experiment 2, the highest uptake of triclopyr occurred where no adjuvant, OS adjuvants, or operational adjuvants were used (Table 1). There was significantly lower uptake of triclopyr where an MO adjuvant was used at either 0.5% or 1.0%. Combining data from Experiments 1 and 2 clearly demonstrated the consistent rate of decline in triclopyr uptake as MO concentration increased (Figure 3).

Based on the results from Experiments 1 and 2, the MO treatments were removed from Experiment 3 and replaced with two concentrations of an MSO adjuvant. In Experiment 3, the highest uptake of triclopyr was achieved with the MSO treatments but uptake was not significantly different to any of the other treatments other than the low rate of OS, where uptake decreased. As with the previous experiments, the adjuvants used operationally did not significantly increase or decrease the uptake of triclopyr relative to the formulated product applied alone.

**FIGURE 4:**
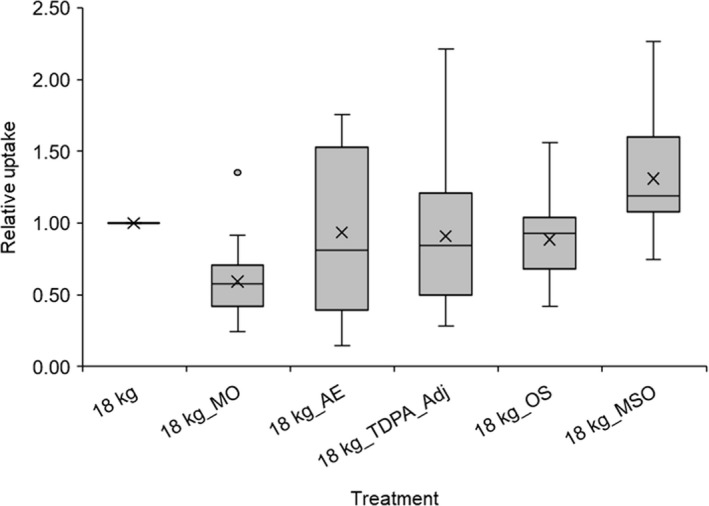
Uptake of triclopyr applied at 18 kg a.i. ha^−1^ in 400 L water (18 kg_), formulated as Grazon^®^, with inclusion of adjuvants in the mix shown as relative to that where no adjuvant was used. Data across experiments (Table 1) were combined where the same adjuvant was tested in more than one experiment. 18 kg = triclopyr applied at 18 kg a.i. ha^−1^ in 400 L water, formulated as Grazon^®^; 18 kg_MO = +mineral oil adjuvant (0.25, 0.5 and 1%); 18 kg_AE = + alcohol ethoxylate adjuvant (0.05 and 0.10%);18 kg_ OS = + organosilicone adjuvant (0.15 and 0.25%); 18 kg_MSO = + methylated seed oil adjuvant (0.5 and 1.0%) and 18 kg_TDPA_Adj = adjuvants used in the operational TDPA mix (20 L/ha MSO, 0.5 L/ha lecithin blend, 4 kg/ha ammonium sulfate)

Normalizing all data to the base treatment of 18 kg a.i. ha^−1^, triclopyr applied in Grazon^®^ with no adjuvant allowed a clearer comparison of the relative effect of adjuvants on uptake across the three experiments (Figure 4). This comparison indicated that the only adjuvant to increase the uptake of triclopyr applied as the formulated product (Grazon^®^) was the MSO.

### 3.4 The relationship between needle injury, uptake, and translocation of triclopyr

As in previous experiments large variation in replicate trees was evident. Triclopyr, applied at 18 kg a.i.ha^−1^ in the equivalent of 400 L water (or 4.5% concentration), was moderately absorbed by foliage (39.8%) within 1 DAT, with 83% of total uptake (7 DAT) occurring within 24 hr (Table 2). By 7 DAT 48% of the amount applied was absorbed. The addition of 20 L (5%) MSO to the carrier increased the rate of uptake within 1 DAT; however, by 7 DAT uptake was not significantly different to that where no adjuvant was used (Table 2). By comparison, uptake at 1 DAT and 7 DAT was significantly lowered where the adjuvants used in the operational TDPA mix were used (DF = 2; *F*‐value = 4.5, *p* = .024; Table 3). Translocation of absorbed triclopyr was highest (91%) where no adjuvants were used, with a statistically similar high rate of translocation (77%) recorded where an MSO was used (Table 2). Translocation of absorbed triclopyr was significantly lower (46%) where triclopyr was applied together with the adjuvants used in the operational mix. No injury to needles was observed when triclopyr was applied alone (Table 2). However, adjuvants significantly increased injury, with most of this apparent within 1 DAT. Lower translocation of absorbed triclopyr was associated with higher needle injury, particularly where the operational adjuvants were used.

### 3.5 THe effect of concentration and adjuvants on needle injury and uptake of dicamba

Uptake of dicamba, applied at 5 kg a.i. ha^−1^, was slow and reached 31% at 7 DAT (Table 3). Doubling the dicamba concentration halved the uptake at 7 DAT to 14.3%, effectively delivering the same dose into the plant. Use of MSO at the equivalent of 20 L/ha, or 5%, significantly increased the rate and total amount of uptake of dicamba, as did the adjuvants used in the operational TDPA mix (Table 3). Both of these treatments more than doubled the uptake of dicamba, relative to that where it was applied alone at 5 kg a.i. ha^−1^. All treatments where adjuvants were used increased needle injury. Needle injury was low when dicamba was applied alone at either 5 kg a.i. ha^−1^ or 10 kg a.i. ha^−1^.

### 3.6 The uptake of triclopyr and dicamba when applied as a tank mix

The uptake of dicamba and triclopyr from individual formulations was similar to that observed in previous experiments, 31% and 26%, respectively, at 7 DAT (Table 4). The addition of triclopyr to dicamba treatments significantly increased dicamba uptake at 7 DAT (Table 4) and the ratio of triclopyr to dicamba had no significant effect on this apparent synergism (Table 4). The addition of dicamba to triclopyr treatments had no significant effect on the uptake of triclopyr (Table 4). The addition of the MSO at 5% significantly increased the uptake of both active ingredients, particularly where triclopyr was applied at a ratio of 3:1 to the rate of dicamba (dicamba + triclopyr + MSO, 5 + 15 + 5%).

Needle injury for all dual‐herbicide treatments was substantially greater than that where herbicides were applied alone, and especially where adjuvants were used (Table 4), as was consistently observed throughout all experiments. The use of the MSO was moderately phytotoxic yet promoted the most herbicide uptake. In earlier studies, such effects were generally not detrimental to triclopyr translocation, but their effects on dicamba movement in the plant, and overall efficacy of treatments were not tested in this study.

## 4 DISCUSSION

The results from these experiments highlighted several factors with respect to the use of triclopyr and dicamba for management of *P. contorta*. Firstly, the spread test is a measure of how well a spray can be expected to cover the plant surface following droplet adhesion. *Pinus contorta* foliage is classified as moderately easy‐to‐wet with the inside surface of the fascicle easier to wet than the waxier, outer fascicle surface (Forster et al., 2014), as was illustrated in this study (Figure 1). In all experiments, treatments were randomly allocated to needle surface and there was no attempt to account for differences in uptake across different surfaces. There is clearly potential to improve treatment spread substantially on the outer needle surfaces, but the effect of that on spray adhesion and uptake into *P. contorta*, and ultimately efficacy, is unknown.

### 4.1 Effect of concentration on uptake

One of the factors that effects foliar uptake of active ingredients is the concentration in the spray mix (Wang & Liu, 2007). While concentration is expected to have an important influence on foliar uptake, its effect on uptake of many agrochemicals is largely unknown (Wang & Liu, 2007). Furthermore, its effect on uptake can be dependent on droplet size and number (Huang, Campbell, Studens, & Fleming, 2000), an aspect which was not examined in this study. This study indicated that for triclopyr applied as an ester to *P. contorta* needles, uptake generally decreased with increasing concentration, particularly above 3%. This resulted in the highest uptake dose being achieved at the operationally applied 18 kg a.i. ha^−1^ in 400 L total volume water, an outcome that generally supports current operational practice for control of dense infestations of conifers. This outcome contrasts that which has been found for glyphosate, for example, where uptake increased with concentration (Wang & Liu, 2007). In a study that examined absorption and translocation of triclopyr ester in *Populus tremuloides*, Huang et al. (2000) found that absorption and translocation of triclopyr decreased as concentration increased and droplet size decreased. Absorption and translocation also decreased as droplet number decreased and droplet size increased (while concentration was held constant). They attributed decreases in uptake at higher concentrations to contact injury.

### 4.2 Effect of adjuvants on uptake

Adjuvants play an important role in increasing the foliar uptake of herbicides (Wang & Liu, 2007). Uptake and translocation of triclopyr applied as Grazon^®^ in an aqueous carrier in this study were equal to, or in some cases better than, that where adjuvants were added to the formulation. Out of all the adjuvants tested for triclopyr, the MSO applied at between 0.5% and 5% of the carrier volume was the most effective, increasing the rate at which triclopyr was absorbed but not the overall amount, when compared to no adjuvant. The use of adjuvants, notably an MSO at 5% or the adjuvants used in the operational TDPA mix, significantly improved the uptake of dicamba into *P. contorta* needles, more than doubling the rate of uptake as well as the total amount absorbed 7 DAT. High surfactant oil concentrate adjuvants have been shown to enhance both lipophilic herbicides (triclopyr) and hydrophilic (dicamba) herbicides (Wirth & Zollinger, 2018). In separate studies, Tse‐Seng et al. (2009) and Wirth and Zollinger (2018) demonstrated an increase in efficacy of triclopyr and dicamba when applied in field with adjuvants, notably crop oils, included in the tank mix.

All aqueous treatments that included an adjuvant significantly increased needle injury, a factor which may be associated with lower translocation in these treatments, particularly the adjuvants used in the operational TDPA mix which were shown to cause high levels of needle injury. The impact of needle injury caused through herbicide phytotoxicity, and subsequent translocation of active ingredient, on efficacy for control of conifers infield is unknown. Contact injury is the damage caused to leaf tissue after spray droplet contact and is not directly attributable to the active ingredient alone. It can develop rapidly, as was observed in several experiments in this study. Contact injury has been shown to isolate and confine the active ingredient physiologically to the site of penetration, thereby decreasing final efficacy. Forster, Zabkiewicz, Murray, and Zedaker (1997) found lower translocation of triclopyr out of woody species leaves where there was greater or earlier development of contact injury. There is uncertainty regarding the role of contact injury in control of dense infestations of wilding conifers using current operational control methods. Following broadcast spraying of the operational TDPA mix, browning of foliage (up to 100%) occurs in some cases within 1 month of application. The main active ingredients in this mix are triclopyr and dicamba, both of which are synthetic auxins. Typically, these active ingredients mimic the deformative and growth inhibiting effects caused by indole acetic acid at a constant very high concentration in the tissue, a phenomenon described as an auxin overdose. However, these effects are seldom observed in field since total brownout of foliage occurs shortly after spraying, after which needles are shed and trees die. It is possible that severe needle injury is playing a key role in achieving the high efficacy typical of the currently used operational TDPA mix, with uptake and translocation as secondary mechanisms contributing to control. Testing a range of lower doses of active ingredients with similar coverage would enable evaluation of this observation.

While contact injury with high uptake rates of triclopyr was observed in this study, lower uptake into *P. contorta* at higher concentrations of triclopyr may also be due to a precipitate forming on the needle surface. Lipophilic active ingredients have been shown to form a precipitate on leaf surfaces at concentrations above a certain threshold, dependent on the co‐formulants or adjuvants used in the product (Forster & Kimberley, 2015). The crystalline deposit reduces the potential for uptake as penetration from the precipitate can be slow or none at all (Zhu & Lin, 2016). While triclopyr is not a highly lipophilic active ingredient (Log *p* = .42), the high concentrations being tested in this study could represent the threshold concentrations for triclopyr precipitation.

### 4.3 Herbicide interactions

The dual‐label study indicated that application of triclopyr and dicamba together did not negatively affect uptake of either active ingredient, and in fact triclopyr enhanced the uptake of dicamba. Increased uptake was also observed when these two active ingredients were applied together with 5% MSO in the equivalent of 400 L water. This result indicates that there is scope to reduce the amount of adjuvant used in the operational TDPA mix without reducing uptake of either active ingredient (and potentially without compromising efficacy).

Ammonium sulfate is currently included at 2.3 kg a.i. ha^−1^ in the operational TDPA mix. This compound has been shown to increase the efficacy of many weak acid herbicides, including dicamba and picloram, particularly when application is made in hard water, as the sulfate ions bind to the antagonistic cations in the spray solution (Wilson & Nishimoto, 1975;Zollinger et al., 2010). Furthermore, it has been proposed that the ammonium ion increases absorption of weak acid herbicides by increasing their movement across leaf cuticles. However, recent concerns over drift of dicamba in many parts of the United States have highlighted the potential risks of applying dicamba together with ammonium sulfate. When dicamba reacts with spray water above 5.5 pH it forms the anionic form of dicamba which has a low vapor potential; however, below pH 5.5 the dicamba converts to the acid form which has a very high vapor potential (Zollinger et al., 2010). Ammonium sulfate is known to reduce the pH of water (Wilson & Nishimoto, 1975), thereby affecting the volatility of dicamba. Mostly this will not be a problem when spraying wilding pines in remote areas where drift onto neighboring crops is unlikely (and where volatilization may actually contribute to efficacy). However, it would be good practice to reduce the potential for drift, if efficacy can be retained by dropping this compound from the operational TDPA mix. Furthermore, Zollinger et al. (2010) found that use of an MSO increased efficacy of dicamba far more than that of ammonium sulfate. The MSOs increased absorption by dissolving the cuticle and allowing greater penetration of active ingredient than surfactant adjuvants (and petroleum oils; Zollinger et al., 2010).

## 5 CONCLUSIONS

The results indicated that uptake of triclopyr, the key active ingredient used for operational control of wilding conifers, decreased with increasing concentration, with the highest uptake dose achieved at the equivalent of 18 kg a.i. ha^−1^ triclopyr. The only adjuvant to increase the uptake of triclopyr relative to no adjuvant was an MSO. The MSO was also shown to double the uptake of dicamba over that where no adjuvant was used. The adjuvants (2.3 kg NH_4_SO_4_, 20 L MSO, 0.5 L surfactant) used in the operational TDPA mix did not increase the uptake and translocation of triclopyr or dicamba over that where only an MSO was included in the mix. This result indicates that there is potential to decrease the amount of adjuvant used without compromising the uptake of triclopyr or dicamba. Needle injury was observed in all studies, and was associated with slightly lower translocation; however, the role of needle injury in final efficacy is unknown. These results indicate that as a first step field studies should test more simple mixes of active ingredients (dicamba, triclopyr, and MSO) and lower rates at similar high volumes (400 L/ha) in aqueous carriers.

## CONFLICT OF INTEREST

The authors declare no conflict of interest.

## AUTHOR CONTRIBUTIONS

All authors made substantial contributions to the design, analysis, and interpretation of data, and were involved in drafting the manuscript for publication.

## References

[pei310012-bib-0001] Davenhill, N. A. , & Preest, N. S. (1974). Interim evaluation of several soil sterilents for the control of contorta pine. Proceedings of the 27th N.Z. Weed and Pest Control Conference. 19–23.

[pei310012-bib-0002] Engelmark, O. , Sjöberg, K. , Andersson, B. , Rosvall, O. , Ågren, G. I. , Baker, W. L. , … Sykes, M. T. (2001). Ecological effects and management aspects of an exotic tree species: The case of lodgepole pine in Sweden. Forest Ecology and Management, 141, 3–13. 10.1016/S0378-1127(00)00498-9

[pei310012-bib-0003] Forster, W. A. , & Kimberley, M. O. (2015). The contribution of spray formulation component variables to foliar uptake of agrichemicals. Pest Management Science, 71, 1324–1334. 10.1002/ps.3934 25354847

[pei310012-bib-0004] Forster, W. A. , Pathan, A. K. , Kimberley, M. O. , Steele, K. D. , & Gaskin, R. E. (2014). The relative influence of retention, uptake, and translocation on the bioefficacy of glyphosate. ACS Symposium Series, 1171, 111–139. 10.1021/bk-2014-1171.ch006

[pei310012-bib-0005] Forster, W. A. , Zabkiewicz, J. A. , Murray, R. J. , & Zedaker, S. M. (1997). Contact phytotoxicity of triclopyr formulations on three plant species in relation to their uptake and translocation. Proceedings of the 50th New Zealand Plant Protection Conference. 125–128.

[pei310012-bib-0007] Gous, S. , Raal, P. , & Watt, M. S. (2014a) Aerial spot treatment using an oil carrier to apply ester based herbicides for control of *Pinus contorta* and *P. nigra* in New Zealand. New Zealand Journal of Forestry Science, 44, 10.1186/s40490-014-0023-6

[pei310012-bib-0008] Gous, S. , Raal, P. , & Watt, M. S. (2014b). Dense wilding conifer control with aerially applied herbicides in New Zealand. New Zealand Journal of Forestry Science, 44, 4. 10.1186/1179-5395-44-4

[pei310012-bib-0009] Gous, S. , Raal, P. , & Watt, M. S. (2015) The evaluation of aerially applied triclopyr mixtures for the control of dense infestations of wilding Pinus contorta in New Zealand. New Zealand Journal of Forestry Science, 45, 10.1186/s40490-014-0031-6

[pei310012-bib-0010] Gous, S. F. , Watt, M. S. , Richardson, B. , & Kimberley, M. O. (2010). Herbicide screening trial to control dormant wilding *Pinus contorta*, *P. mugo* and *Pseudotsuga menziesii* during winter. New Zealand Journal of Forestry Science, 40, 153–159.

[pei310012-bib-0011] Huang, J. Z. , Campbell, R. A. , Studens, J. A. , & Fleming, R. A. (2000). Absorption and translocation of triclopyr ester in *Populus tremuloides* . Weed Science, 48, 680–687. 10.1614/0043-1745(2000)048[0680:aatote]2.0.co;2

[pei310012-bib-0012] Langer, E. R. (1992). Chemical control of wilding conifer seedlings in New Zealand. Plant Protection Quarterly, 7, 135–139.

[pei310012-bib-0013] Ledgard, N. (2001). The spread of lodgepole pine (*Pinus contorta*, Dougl.) in New Zealand. Forest Ecology and Management, 141, 43–57. 10.1016/s0378-1127(00)00488-6

[pei310012-bib-0014] Ledgard, N. J. (2008). Assessing the risk of the natural regeneration of introduced conifers, or wilding spread. New Zealand Plant Protetcion, 61, 91–97. 10.30843/nzpp.2008.61.6877

[pei310012-bib-0016] McBean, C. (2012). The pesticide manual (16th ed.). Hampshire, UK: British Crop Protection Council.

[pei310012-bib-0017] Ministry for Primary Industries . (2015). New Zealand Wilding Conifer Management Strategy 2015–2030. Wellington, New Zealand: Ministry for Primary Industries.

[pei310012-bib-0018] Nairn, J. J. , Forster, W. A. , & van Leeuwen, R. M. (2016). Effect of solution and leaf surface polarity on droplet spread area and contact angle. Pest Management Science, 72, 551–557. 10.1002/ps.4022 25864426

[pei310012-bib-0019] National Wilding Conifer Programme . (2018). Aerial foliar spray application (AFSA). Wellingtion, New Zealand: Better Biosecurity Solutions.

[pei310012-bib-0020] Preest, D. S. (1985). Chemical aids to planting site preparation. Rotorua, New Zealand: Forest Research Institute, New Zealand Forest Service.

[pei310012-bib-0021] Ray, J. W. , & Davehill, N. A. (1991). Evaluation of herbicides for the control of Pinus contorta.Proceedings of the 44th New Zealand Weed and Pest Control Conference. 21–24.

[pei310012-bib-0022] Rouget, M. , Richardson, D. M. , Milton, S. J. , & Polakow, D. (2004). Predicting invasion dynamics of four alien Pinus species in a highly fragmented semi‐arid shrublands in South Africa. Plant Ecology, 152, 79–92.

[pei310012-bib-0023] Simberloff, D. , Martin, J.‐L. , Genovesi, P. , Maris, V. , Wardle, D. A. , Aronson, J. , … Vilà, M. (2013). Impacts of biological invasions: What's what and the way forward. Trends in Ecology & Evolution, 28, 58–66. 10.1016/j.tree.2012.07.013 22889499

[pei310012-bib-0024] Stock, D. , Edgerton, B. M. , Gaskin, R. E. , & Holloway, P. J. (1992). Surfactant‐enhanced foliar uptake of some organic compounds: Interactions with two model poloxyethylene aliphatitic alcohols. Pesticide Science, 34, 233–242. 10.1002/ps.2780340308

[pei310012-bib-0025] Tse‐Seng, C. , Kaben, A. M. , & Thye‐San, C. (2009). Proper adjuvant selection to enhance the activity of triclopyr combined with metsulfuron on the control of Hedyotis vertillata. Weed Biology and Management, 9, 179–184. 10.1111/j.1445-6664.2009.00337.x

[pei310012-bib-0026] Vanhaecke, M. (2000). Agrochemical adjuvant: Mode of action and benefits. Planters, 76, 123–136.

[pei310012-bib-0027] Wang, C. J. , & Liu, Z. Q. (2007). Foliar uptake of pesticides‐Present status and future challenges. Pesticide Biochemistry and Physiology, 87, 1–8. 10.1016/j.pestbp.2006.04.004

[pei310012-bib-0028] Wilson, B. J. , & Nishimoto, R. K. (1975). Ammonium sulfate enhancement of picloram absorption and activity. Weed Science, 23, 289–295.

[pei310012-bib-0029] Wirth, D. , & Zollinger, R. K. (2018). Increase in herbicide efficacy using high surfactant oil concentrate adjuvants. Pesticide Formulation and Delivery Systems. 10.1520/STP160220170017

[pei310012-bib-0030] Zhu, H. , & Lin, J. (2016). Coverage area and fading time of surfactant‐amended herbicidal droplets on cucurbitaceous leaves. Transactions of the ASABE, 59, 829–838. 10.13031/trans.59.11427

[pei310012-bib-0031] Zollinger, R. K. , Nalewaja, J. D. , Peterson, D. E. , Young, B. G. , Lindsay, A. D. , Zollinger, R. , & Dean, S. W. (2010). Effect of hard water and ammonium sulfate on weak acid herbicide activity. Journal of ASTM International, 7. 10.1520/JAI102869

